# (*Z*)-Benzyl 2-(5-methyl-2-oxoindolin-3-yl­idene)hydrazinecarbodi­thio­ate

**DOI:** 10.1107/S2414314623007824

**Published:** 2023-09-12

**Authors:** Mohd Abdul Fatah Abdul Manan, David B. Cordes, Aidan P. McKay, Mohd Fazli Mohammat, Mohd Fadhlizil Fasihi Mohd Aluwi, Nor Saliyana Jumali

**Affiliations:** a Faculty of Applied Sciences, Universiti Teknologi MARA, 40450 Shah Alam, Selangor, Malaysia; b EaStCHEM School of Chemistry, University of St Andrews, St Andrews, Fife KY16 9ST, United Kingdom; c Organic Synthesis Research Laboratory, Institute of Science, Universiti Teknologi MARA, 42300 Bandar Puncak Alam, Selangor, Malaysia; d Centre for Bio-aromatic Research, Universiti Malaysia Pahang, Lebuhraya Tun Razak, 26300 Gambang, Kuantan, Pahang, Malaysia; e Department of Chemistry, Kuliyyah of Science, International Islamic University Malaysia, 25200 Bandar Indera Mahkota, Kuantan, Pahang, Malaysia; University of Aberdeen, United Kingdom

**Keywords:** crystal structure, di­thio­carbazate, 5-methyl­isatin, *Z* configuration, hydrogen bonding

## Abstract

The title compound, C_17_H_15_N_3_OS_2_ was obtained from the condensation reaction of *S*-benzyl­dithio­carbazate and 5-methyl­isatin. In the solid-state, the mol­ecule adopts a *Z* configuration with the 5-methyl­isatin and di­thio­carbazate groups located on the same side of the C=N bond, involving an intra­molecular N—H⋯O hydrogen bond.

## Abstract 2

The crystal structure of a new di­thio­carbazate imine, obtained from the condensation reaction of *S*-benzyl­dithio­carbazate and 5-methyl­isatin, is described.

## Graphical abstract

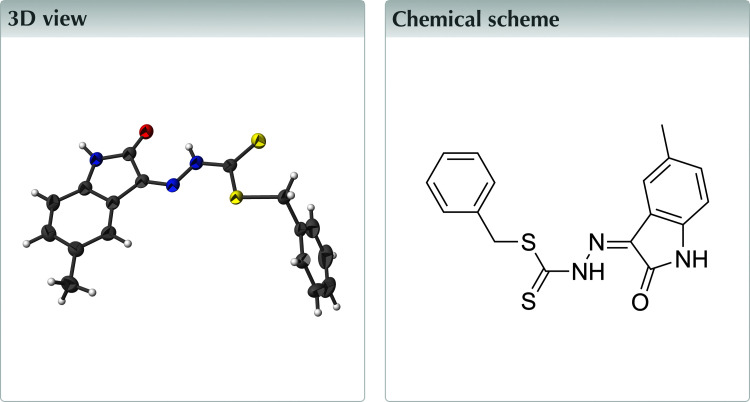

## Structure description

Di­thio­carbazate-based imines and some of their metal complexes possess diverse biological applications (*e.g.*, Manan & Cordes, 2022 ▸). In addition, the applications of these compounds have evolved in research areas such as semiconductor devices (Irfan *et al.*, 2020 ▸) and the photocatalytic production of hydrogen (Wise *et al.*, 2015 ▸). In a contin­uation of our previous work on isatin-based imines derived from di­thio­carbazate compounds (Manan *et al.*, 2011 ▸), the title compound was synthesized and its crystal structure is reported herein.

**Figure 1:**
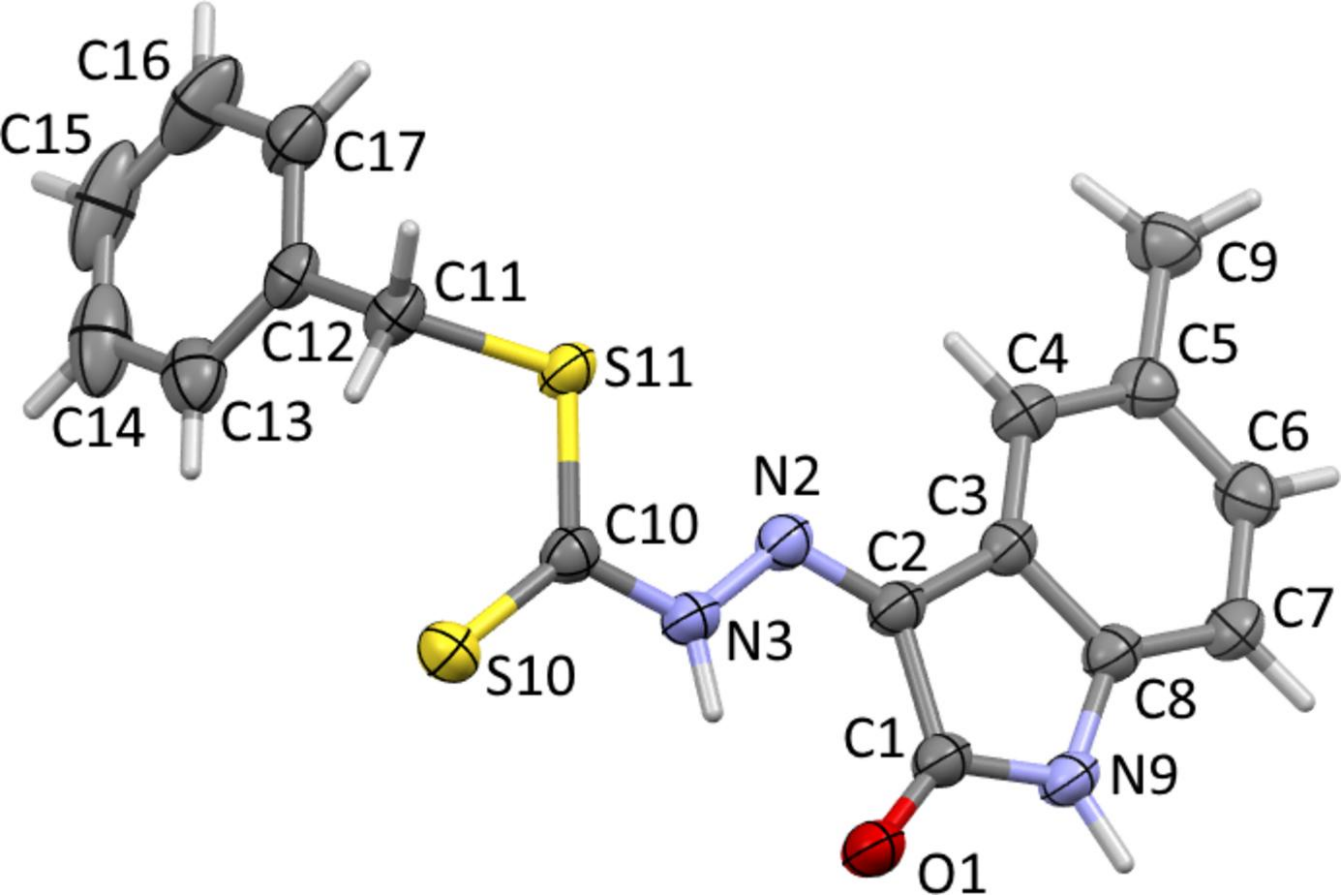
The mol­ecular structure of the title compound, showing displacement ellipsoids drawn at the 50% probability level.

The title compound, C_17_H_15_N_3_OS_2_ crystallizes in the triclinic space group *P* 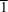 with one mol­ecule in asymmetric unit. The structure is present as the thio­amide tautomer and in the *Z* isomeric form (Fig. 1 ▸) as a consequence of the formation of an intra­molecular N3—H3⋯O1 hydrogen bond (Table 1 ▸). The C10=S10 and C10—S11 lengths of 1.6544 (16) and 1.7449 (16) Å, respectively, are comparable to those reported for *S*-benzyl 3–2(bromo­benzyl­idene)di­thio­carbazate (Qiu & Luo, 2007 ▸), benzyl 3-(3,4,5-tri­meth­oxy­benzyl­idene)di­thio­carbazate (Islam *et al.*, 2016 ▸) and benzyl 3-(10-oxo-9,10-di­hydro­phenanthren-9-yl­idene)di­thio­carbazate (Liu *et al.*, 2009 ▸). The observed C—S bond lengths are both inter­mediate between reference values of 1.82 Å for a C—S single bond and 1.56 Å for a C=S double bond (Tarafder *et al.*, 2002 ▸), indicative of conjugation effects through the π-system. As a result of the delocalization of electrons in the 5-methyl­isatin ring, the N2—N3 bond distance of 1.3509 (19) Å is slightly shorter than the corresponding bond in the unsubstituted precursor compound (Shanmuga Sundara Raj *et al.*, 2000 ▸).

The central CN_2_S_2_ residue in the title compound is close to planar (r.m.s deviation = 0.052 Å) and forms dihedral angles of 9.34 (3) and 72.80 (5)° with the substituted benzyl and 5-methyl­isatin rings, respectively, indicating a highly twisted mol­ecule; the dihedral angle between the rings is 70.87 (5)°. The N2—N3—C10—S10 fragment adopts an *anti* conformation with a torsion angle of 174.23 (11)°, while the N2—N3—C10—S11 fragment is *syn* with a torsion angle of −6.67 (19)°. This conformation is similar to those of three closely related compounds benzyl 2-(5-chloro-2-oxo-1,2-di­hydro-3*H*-indol-3-yl­idene)hydrazinecarbodi­thio­ate, benzyl 2-(5-fluoro-2-oxo-1,2-di­hydro-3*H*-indol-3-yl­idene)hydrazine­carbodi­thio­ate and benzyl 2-(5-bromo-2-oxo-1,2-di­hydro-3*H*-indol-3-yl­idene)hydrazinecarbodi­thio­ate (Manan *et al.*, 2011 ▸).

**Figure 2:**
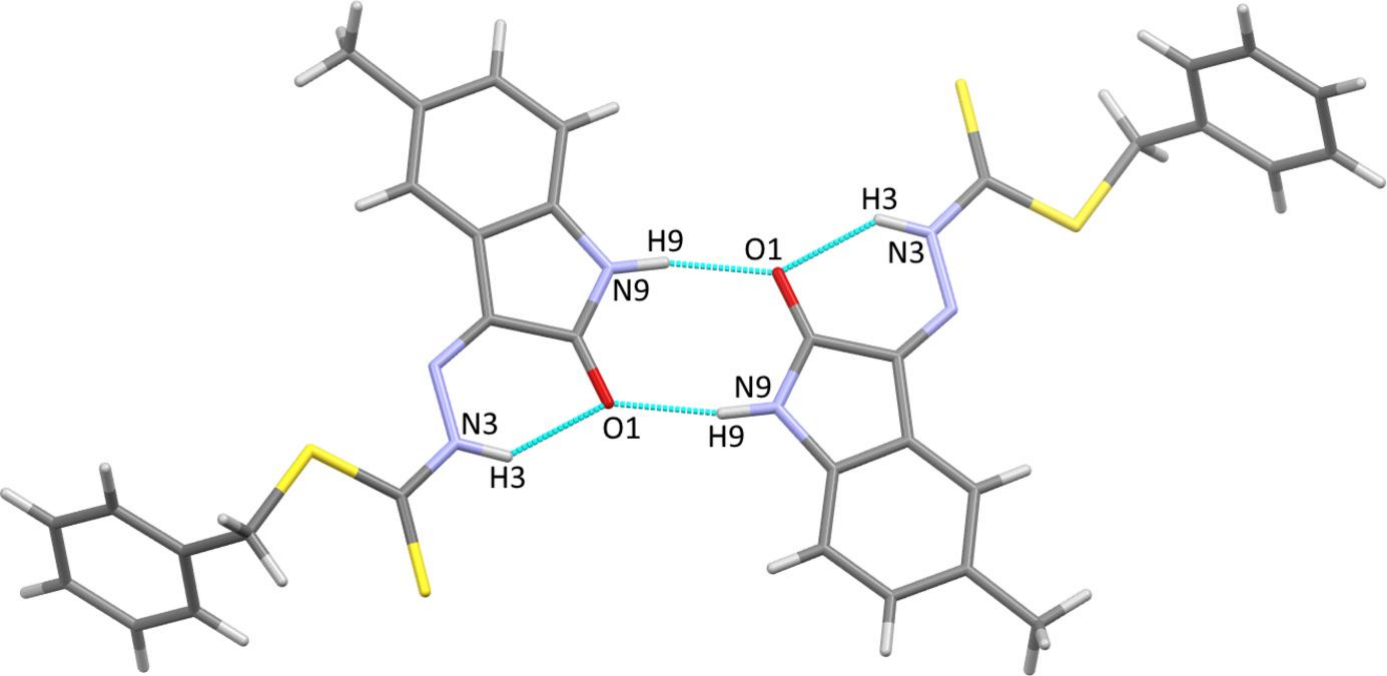
View of a hydrogen-bonded dimer of the title compound showing both intra­molecular and inter­molecular N—H⋯O hydrogen bonds. The right-hand mol­ecule is generated by the symmetry operation 2 − *x*, −*y*, 1 − *z*.

In the crystal, the title compound forms inversion dimers joined by pairs of N9—H9⋯O1 hydrogen bonds (Fig. 2 ▸, Table 1 ▸) in the common 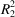 (8) motif (Bernstein *et al.*, 1995 ▸). The dimers then pack into sheets propagating in the (001) plane through carbonyl-to-π [O⋯centroid distance = 3.418 (2) Å] and C—H⋯π [H⋯centroid distance = 3.142 (1) Å, C⋯centroid distance = 3.846 (2) Å] inter­actions. Equivalent dimers are observed in the 5-bromo and 5-chloro compounds mentioned above, as well as in 2-(5-nitro-2-oxo-1,2-di­hydro-3*H*-indol-3-yl­idene)hydrazinecarbodi­thio­ate (Pereira *et al.*, 2021 ▸) and the parent compound 2-(2-oxo-1,2-di­hydro-3*H*-indol-3-yl­idene)hydrazinecarbodi­thio­ate (Ali *et al.*, 2011 ▸). The aceto­nitrile solvate of the parent compound (Ali *et al.*, 2011 ▸) does not form dimers and instead forms discrete N—H⋯N hydrogen bonds to the solvate. Unlike the majority of related compounds, the 5-fluoro compound (Manan *et al.*, 2011 ▸) does not form dimers and instead packs through strong imine to π inter­actions (centroid⋯centroid separation = 3.213 Å), with weaker N—H⋯S=C hydrogen bonds involving the amide site.

## Synthesis and crystallization

The di­thio­carbazate precursor, SBDTC was prepared by a literature method (Ali & Tarafder, 1977 ▸). The title compound was prepared by adding 5-methyl­isatin (1.61 g, 10.0 mmol, 1.0 eq) dissolved in hot ethanol (10 ml), to a solution of the precursor, SBDTC (1.98 g, 10.0 mmol, 1.0 e.q) in hot ethanol (35 ml). The mixture was heated (80°C) with continuous stirring for 15 min and later allowed to stand for about 20 min at room temperature until a precipitate was formed, which was then filtered and dried over silica gel, yielding orange crystals on recrystallization from ethanol solution (yield: 2.73 g, 80%). m.p. 216–217°C; ^1^H (400 MHz, *d*_6_-DMSO) δ: (p.p.m): 2.26 (3*H*, *s*), 4.52 (2*H*, *s*), 6.82–7.45 (8*H*, *m*), 11.26 (1*H*, *s*), 13.94 (1*H*, *s*); GCMS: [*M*]^+^ at *m*/*z* 341.

## Refinement

Crystal data, data collection and structure refinement details are summarized in Table 2 ▸.

## Supplementary Material

Crystal structure: contains datablock(s) I. DOI: 10.1107/S2414314623007824/hb4449sup1.cif

Structure factors: contains datablock(s) I. DOI: 10.1107/S2414314623007824/hb4449Isup2.hkl

Supporting information file. DOI: 10.1107/S2414314623007824/hb4449Isup3.mol

Supporting information file. DOI: 10.1107/S2414314623007824/hb4449Isup4.cml

CCDC reference: 2293455

Additional supporting information:  crystallographic information; 3D view; checkCIF report

## Figures and Tables

**Table 1:** Hydrogen-bond geometry (Å, °)

*D*—H⋯*A*	*D*—H	H⋯*A*	*D*⋯*A*	*D*—H⋯*A*
N3—H3⋯O1	0.91 (2)	2.00 (2)	2.7539 (17)	139 (2)
N9—H9⋯O1^i^	0.94 (2)	1.91 (2)	2.8341 (18)	166 (2)

Symmetry code: (i) 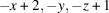 .

**Table 2:** Experimental details

Crystal data	
Chemical formula	C_17_H_15_N_3_OS_2_
*M*_r_	341.44
Crystal system, space group	Triclinic, *P* 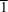
Temperature (K)	173
*a*, *b*, *c* (Å)	6.5733 (2), 8.0601 (2), 15.8280 (4)
α, β, γ (°)	95.442 (2), 99.527 (2), 90.360 (2)
*V* (Å^3^)	823.09 (4)
*Z*	2
Radiation type	Cu *K*α
μ (mm^−1^)	2.99
Crystal size (mm)	0.12 × 0.09 × 0.02

Data collection	
Diffractometer	Rigaku XtaLAB P100K
Absorption correction	Multi-scan (*CrysAlis PRO*; Rigaku OD, 2023 ▸)
*T*_min_, *T*_max_	0.748, 1.000
No. of measured, independent and observed [*I* > 2σ(*I*)] reflections	14207, 2876, 2623
*R*_int_	0.034
(sin θ/λ)_max_ (Å^−1^)	0.595

Refinement	
*R*[*F* ^2^ > 2σ(*F* ^2^)], *wR*(*F* ^2^), *S*	0.034, 0.097, 1.07
No. of reflections	2876
No. of parameters	217
No. of restraints	2
H-atom treatment	H atoms treated by a mixture of independent and constrained refinement
Δρ_max_, Δρ_min_ (e Å^−3^)	0.38, −0.18

Computer programs: *CrysAlis PRO* (Rigaku OD, 2023 ▸), *SHELXT2018/2* (Sheldrick, 2015*a* ▸), *SHELXL2018/3* (Sheldrick, 2015*b* ▸) and *OLEX2* (Dolomanov *et al.*, 2009 ▸).

## full crystallographic data

### Crystal data

**Table:**

C_17_H_15_N_3_OS_2_	*Z* = 2
*M**_r_* = 341.44	*F*(000) = 356
Triclinic, *P*1	*D*_x_ = 1.378 Mg m^−^^3^
*a* = 6.5733 (2) Å	Cu *K*α radiation, λ = 1.54184 Å
*b* = 8.0601 (2) Å	Cell parameters from 8603 reflections
*c* = 15.8280 (4) Å	θ = 2.8–66.0°
α = 95.442 (2)°	µ = 2.99 mm^−^^1^
β = 99.527 (2)°	*T* = 173 K
γ = 90.360 (2)°	Plate, yellow
*V* = 823.09 (4) Å^3^	0.12 × 0.09 × 0.02 mm

### Data collection

**Table:**

Rigaku XtaLAB P100K diffractometer	2876 independent reflections
Radiation source: Rotating Anode, Rigaku MM-007HF	2623 reflections with *I* > 2σ(*I*)
Rigaku Osmic Confocal Optical System monochromator	*R*_int_ = 0.034
Detector resolution: 5.8140 pixels mm^-1^	θ_max_ = 66.5°, θ_min_ = 2.8°
shutterless scans	*h* = −7→7
Absorption correction: multi-scan (CrysAlisPro; Rigaku OD, 2023)	*k* = −9→9
*T*_min_ = 0.748, *T*_max_ = 1.000	*l* = −18→18
14207 measured reflections	

### Refinement

**Table:**

Refinement on *F*^2^	Primary atom site location: dual
Least-squares matrix: full	Hydrogen site location: mixed
*R*[*F*^2^ > 2σ(*F*^2^)] = 0.034	H atoms treated by a mixture of independent and constrained refinement
*wR*(*F*^2^) = 0.097	*w* = 1/[σ^2^(*F*_o_^2^) + (0.0626*P*)^2^ + 0.1676*P*] where *P* = (*F*_o_^2^ + 2*F*_c_^2^)/3
*S* = 1.07	(Δ/σ)_max_ < 0.001
2876 reflections	Δρ_max_ = 0.38 e Å^−^^3^
217 parameters	Δρ_min_ = −0.17 e Å^−^^3^
2 restraints	

### Special details

**Table:**

Geometry. All esds (except the esd in the dihedral angle between two l.s. planes) are estimated using the full covariance matrix. The cell esds are taken into account individually in the estimation of esds in distances, angles and torsion angles; correlations between esds in cell parameters are only used when they are defined by crystal symmetry. An approximate (isotropic) treatment of cell esds is used for estimating esds involving l.s. planes.
Refinement. Carbon-bound H atoms were included in calculated positions (C—H distances are 0.98 Å for methyl H atoms, 0.99 Å for methylene H atoms 0.95 Å for phenyl H atoms) and refined as riding atoms with *U*_iso_(H) = 1.2 *U*_eq_(parent atom, methylene and phenyl H atoms) or *U*_iso_(H) = 1.5 *U*_eq_(parent atom, methyl H atoms). Nitrogen-bound hydrogen atoms were located from the difference Fourier map and refined isotropically subject to a distance restraint.

### Fractional atomic coordinates and isotropic or equivalent isotropic displacement parameters (Å^2^)

**Table:**

	*x*	*y*	*z*	*U*_iso_*/*U*_eq_	
S11	0.21331 (6)	0.33788 (5)	0.21855 (2)	0.03493 (14)	
S10	0.58661 (6)	0.23720 (6)	0.13457 (3)	0.04020 (15)	
O1	0.86838 (18)	0.08666 (16)	0.40267 (8)	0.0398 (3)	
N3	0.5546 (2)	0.22521 (17)	0.29604 (9)	0.0341 (3)	
N2	0.4614 (2)	0.26067 (17)	0.36534 (9)	0.0326 (3)	
N9	0.7901 (2)	0.11126 (18)	0.54096 (9)	0.0358 (3)	
C12	0.1188 (3)	0.5709 (2)	0.10260 (10)	0.0337 (3)	
C1	0.7576 (2)	0.1314 (2)	0.45642 (11)	0.0335 (3)	
C2	0.5567 (2)	0.22039 (19)	0.43850 (10)	0.0325 (3)	
C10	0.4621 (2)	0.26451 (19)	0.21733 (10)	0.0319 (3)	
C8	0.6299 (2)	0.1807 (2)	0.58149 (11)	0.0340 (3)	
C3	0.4846 (2)	0.24993 (19)	0.52025 (10)	0.0324 (3)	
C5	0.2894 (3)	0.3413 (2)	0.63055 (11)	0.0378 (4)	
C11	0.1296 (3)	0.3858 (2)	0.10785 (10)	0.0359 (4)	
H11A	0.227507	0.338283	0.071281	0.043*	
H11B	−0.007988	0.333708	0.085640	0.043*	
C4	0.3146 (3)	0.3301 (2)	0.54485 (11)	0.0365 (4)	
H4	0.216108	0.376892	0.503346	0.044*	
C7	0.6075 (3)	0.1888 (2)	0.66702 (11)	0.0394 (4)	
H7	0.705150	0.140761	0.708391	0.047*	
C6	0.4357 (3)	0.2702 (2)	0.69000 (11)	0.0415 (4)	
H6	0.417242	0.277589	0.748510	0.050*	
C17	−0.0561 (3)	0.6540 (2)	0.11867 (12)	0.0452 (4)	
H17	−0.169973	0.593731	0.131545	0.054*	
C13	0.2833 (3)	0.6589 (3)	0.08239 (12)	0.0478 (5)	
H13	0.403301	0.602050	0.070803	0.057*	
C9	0.1083 (3)	0.4309 (3)	0.65976 (12)	0.0474 (4)	
H9A	0.023698	0.475703	0.610469	0.071*	
H9B	0.158867	0.522536	0.703588	0.071*	
H9C	0.024801	0.352666	0.684170	0.071*	
C16	−0.0654 (5)	0.8257 (3)	0.11601 (14)	0.0685 (7)	
H16	−0.184886	0.883158	0.127739	0.082*	
C14	0.2728 (5)	0.8293 (3)	0.07910 (14)	0.0690 (8)	
H14	0.385349	0.889230	0.064934	0.083*	
C15	0.1000 (6)	0.9125 (3)	0.09623 (15)	0.0803 (10)	
H15	0.094106	1.029931	0.094482	0.096*	
H3	0.680 (3)	0.177 (3)	0.3057 (14)	0.052 (6)*	
H9	0.901 (3)	0.053 (3)	0.5684 (14)	0.057 (6)*	

### Atomic displacement parameters (Å^2^)

**Table:**

	*U*^11^	*U*^22^	*U*^33^	*U*^12^	*U*^13^	*U*^23^
S11	0.0346 (2)	0.0399 (2)	0.0312 (2)	0.01365 (17)	0.00499 (16)	0.00834 (16)
S10	0.0370 (2)	0.0487 (3)	0.0375 (2)	0.00624 (18)	0.01188 (17)	0.00715 (18)
O1	0.0344 (6)	0.0487 (7)	0.0373 (6)	0.0126 (5)	0.0052 (5)	0.0097 (5)
N3	0.0297 (7)	0.0389 (7)	0.0345 (7)	0.0087 (6)	0.0044 (6)	0.0085 (6)
N2	0.0320 (7)	0.0332 (7)	0.0325 (7)	0.0056 (5)	0.0030 (5)	0.0060 (5)
N9	0.0306 (7)	0.0424 (8)	0.0334 (7)	0.0086 (6)	0.0001 (5)	0.0081 (6)
C12	0.0413 (9)	0.0329 (8)	0.0249 (7)	0.0039 (7)	−0.0017 (6)	0.0050 (6)
C1	0.0294 (8)	0.0337 (8)	0.0365 (8)	0.0042 (6)	0.0015 (6)	0.0063 (6)
C2	0.0300 (8)	0.0310 (8)	0.0360 (8)	0.0046 (6)	0.0021 (6)	0.0067 (6)
C10	0.0320 (8)	0.0278 (7)	0.0353 (8)	0.0019 (6)	0.0031 (6)	0.0046 (6)
C8	0.0306 (8)	0.0349 (8)	0.0351 (8)	0.0018 (6)	0.0005 (6)	0.0054 (6)
C3	0.0313 (8)	0.0317 (8)	0.0332 (8)	0.0019 (6)	0.0010 (6)	0.0055 (6)
C5	0.0335 (8)	0.0406 (9)	0.0386 (9)	−0.0002 (7)	0.0056 (7)	0.0010 (7)
C11	0.0403 (9)	0.0351 (8)	0.0303 (8)	0.0067 (7)	−0.0016 (6)	0.0055 (6)
C4	0.0338 (8)	0.0364 (9)	0.0383 (9)	0.0051 (7)	0.0021 (7)	0.0054 (7)
C7	0.0355 (9)	0.0483 (10)	0.0332 (8)	0.0024 (7)	−0.0010 (7)	0.0089 (7)
C6	0.0396 (9)	0.0511 (10)	0.0333 (9)	−0.0013 (8)	0.0056 (7)	0.0019 (7)
C17	0.0529 (11)	0.0452 (10)	0.0376 (9)	0.0157 (8)	0.0049 (8)	0.0083 (7)
C13	0.0509 (10)	0.0534 (11)	0.0367 (9)	−0.0098 (9)	−0.0009 (8)	0.0076 (8)
C9	0.0419 (10)	0.0576 (11)	0.0428 (10)	0.0067 (8)	0.0102 (8)	−0.0013 (8)
C16	0.105 (2)	0.0485 (12)	0.0467 (12)	0.0390 (13)	−0.0009 (12)	0.0012 (9)
C14	0.104 (2)	0.0527 (13)	0.0441 (12)	−0.0332 (14)	−0.0100 (12)	0.0135 (10)
C15	0.155 (3)	0.0294 (10)	0.0451 (12)	−0.0012 (15)	−0.0179 (15)	0.0056 (9)

### Geometric parameters (Å, º)

**Table:**

S11—C10	1.7449 (16)	C5—C6	1.396 (3)
S11—C11	1.8260 (16)	C5—C9	1.510 (3)
S10—C10	1.6544 (16)	C11—H11A	0.9900
O1—C1	1.239 (2)	C11—H11B	0.9900
N3—N2	1.3509 (19)	C4—H4	0.9500
N3—C10	1.360 (2)	C7—H7	0.9500
N3—H3	0.912 (16)	C7—C6	1.392 (3)
N2—C2	1.293 (2)	C6—H6	0.9500
N9—C1	1.345 (2)	C17—H17	0.9500
N9—C8	1.413 (2)	C17—C16	1.390 (3)
N9—H9	0.938 (16)	C13—H13	0.9500
C12—C11	1.503 (2)	C13—C14	1.381 (3)
C12—C17	1.381 (3)	C9—H9A	0.9800
C12—C13	1.387 (2)	C9—H9B	0.9800
C1—C2	1.505 (2)	C9—H9C	0.9800
C2—C3	1.449 (2)	C16—H16	0.9500
C8—C3	1.402 (2)	C16—C15	1.384 (4)
C8—C7	1.381 (2)	C14—H14	0.9500
C3—C4	1.387 (2)	C14—C15	1.375 (4)
C5—C4	1.388 (2)	C15—H15	0.9500
			
C10—S11—C11	103.16 (8)	C12—C11—H11B	109.4
N2—N3—C10	119.92 (14)	H11A—C11—H11B	108.0
N2—N3—H3	116.6 (14)	C3—C4—C5	119.52 (16)
C10—N3—H3	123.4 (14)	C3—C4—H4	120.2
C2—N2—N3	117.17 (14)	C5—C4—H4	120.2
C1—N9—C8	111.27 (14)	C8—C7—H7	121.4
C1—N9—H9	123.9 (15)	C8—C7—C6	117.16 (16)
C8—N9—H9	124.7 (15)	C6—C7—H7	121.4
C17—C12—C11	119.66 (16)	C5—C6—H6	118.7
C17—C12—C13	119.77 (17)	C7—C6—C5	122.66 (16)
C13—C12—C11	120.57 (16)	C7—C6—H6	118.7
O1—C1—N9	127.80 (15)	C12—C17—H17	120.0
O1—C1—C2	125.75 (15)	C12—C17—C16	120.0 (2)
N9—C1—C2	106.46 (14)	C16—C17—H17	120.0
N2—C2—C1	128.01 (15)	C12—C13—H13	120.0
N2—C2—C3	125.68 (15)	C14—C13—C12	120.0 (2)
C3—C2—C1	106.30 (13)	C14—C13—H13	120.0
S10—C10—S11	128.14 (10)	C5—C9—H9A	109.5
N3—C10—S11	112.22 (12)	C5—C9—H9B	109.5
N3—C10—S10	119.64 (13)	C5—C9—H9C	109.5
C3—C8—N9	109.25 (14)	H9A—C9—H9B	109.5
C7—C8—N9	129.31 (15)	H9A—C9—H9C	109.5
C7—C8—C3	121.43 (16)	H9B—C9—H9C	109.5
C8—C3—C2	106.70 (14)	C17—C16—H16	120.1
C4—C3—C2	133.05 (15)	C15—C16—C17	119.8 (2)
C4—C3—C8	120.24 (16)	C15—C16—H16	120.1
C4—C5—C6	118.99 (16)	C13—C14—H14	119.9
C4—C5—C9	120.83 (16)	C15—C14—C13	120.3 (2)
C6—C5—C9	120.18 (16)	C15—C14—H14	119.9
S11—C11—H11A	109.4	C16—C15—H15	119.9
S11—C11—H11B	109.4	C14—C15—C16	120.1 (2)
C12—C11—S11	111.12 (11)	C14—C15—H15	119.9
C12—C11—H11A	109.4		
			
O1—C1—C2—N2	2.5 (3)	C8—N9—C1—O1	179.33 (16)
O1—C1—C2—C3	−178.95 (16)	C8—N9—C1—C2	−0.72 (18)
N3—N2—C2—C1	−2.7 (2)	C8—C3—C4—C5	0.0 (2)
N3—N2—C2—C3	178.99 (14)	C8—C7—C6—C5	0.2 (3)
N2—N3—C10—S11	−6.67 (19)	C3—C8—C7—C6	−0.7 (3)
N2—N3—C10—S10	174.23 (11)	C11—S11—C10—S10	−2.51 (13)
N2—C2—C3—C8	177.57 (15)	C11—S11—C10—N3	178.48 (11)
N2—C2—C3—C4	−3.7 (3)	C11—C12—C17—C16	178.43 (17)
N9—C1—C2—N2	−177.48 (15)	C11—C12—C13—C14	−178.98 (16)
N9—C1—C2—C3	1.09 (17)	C4—C5—C6—C7	0.4 (3)
N9—C8—C3—C2	0.65 (18)	C7—C8—C3—C2	179.54 (15)
N9—C8—C3—C4	−178.28 (14)	C7—C8—C3—C4	0.6 (2)
N9—C8—C7—C6	177.93 (16)	C6—C5—C4—C3	−0.5 (3)
C12—C17—C16—C15	0.8 (3)	C17—C12—C11—S11	−84.17 (17)
C12—C13—C14—C15	0.3 (3)	C17—C12—C13—C14	0.5 (3)
C1—N9—C8—C3	0.07 (19)	C17—C16—C15—C14	0.1 (3)
C1—N9—C8—C7	−178.72 (17)	C13—C12—C11—S11	95.35 (16)
C1—C2—C3—C8	−1.05 (17)	C13—C12—C17—C16	−1.1 (3)
C1—C2—C3—C4	177.68 (17)	C13—C14—C15—C16	−0.7 (3)
C2—C3—C4—C5	−178.56 (16)	C9—C5—C4—C3	178.71 (16)
C10—S11—C11—C12	−109.04 (13)	C9—C5—C6—C7	−178.83 (16)
C10—N3—N2—C2	179.99 (14)		

### Hydrogen-bond geometry (Å, º)

**Table:**

*D*—H···*A*	*D*—H	H···*A*	*D*···*A*	*D*—H···*A*
N3—H3···O1	0.91 (2)	2.00 (2)	2.7539 (17)	139 (2)
N9—H9···O1^i^	0.94 (2)	1.91 (2)	2.8341 (18)	166 (2)

Symmetry code: (i) −*x*+2, −*y*, −*z*+1.
